# Impact of COVID-19 on antidepressant prescription: a matched cohort study using the National insurance claims database in Japan

**DOI:** 10.1186/s12888-025-07172-w

**Published:** 2025-07-25

**Authors:** Daisuke Miyamori, Shuhei Yoshida, Wataru Omori, Saori Kashima, Masanori Ito

**Affiliations:** 1 Department of General Internal Medicine, Hiroshima University Hospital, 1-2-3, Kasumi Minami-ku, Hiroshima-Shi, Hiroshima, 734-8551 Japan; 2 Department of Psychiatry and Neurosciences, Graduate School of Biomedical Sciences, Hiroshima University, Hiroshima, Japan; 3 Center for the Planetary Health and Innovation Science, The IDEC Institute, Hiroshima University, Hiroshima, Japan; 4 Environmental Health Sciences Laboratory, Graduate School of Advanced Science and Engineering, Hiroshima University, Hiroshima, Japan

**Keywords:** COVID-19, Antidepressants, National insurance claims database, Matched cohort study, Depression, SARS-CoV-2

## Abstract

**Background:**

The COVID-19 pandemic has significantly affected global health, leading to an increased incidence of mental health disorders, particularly depression.

**Methods:**

This matched cohort study aimed to assess the impact of COVID-19 on antidepressant prescriptions using data from Japan’s National Insurance Claims Database. The primary outcome was new antidepressant prescriptions, with SARS-CoV-2 infection as exposure. Data were matched by age, sex, Charlson comorbidity index (CCI), and insurance enrollment date to compare SARS-CoV-2 infected individuals with matched uninfected controls. Follow-up was terminated upon new antidepressant prescriptions or at the end of the study. The incidence rate ratios (IRR) and differences were calculated and compared using survival analysis.

**Results:**

In this study, 16 million participants were analyzed, forming approximately 2.5 million pairs. Over 34 months (median follow-up: 7 months, interquartile range 4–13), there were 54,352 and 33,101 antidepressant prescriptions in the COVID-19 and control groups, respectively, with a cumulative incidence difference of 841 events (95% confidence interval [CI]: 815–860) per 1,000,000 person-months and an IRR of 1.56 (95%CI 1.54–1.58). The largest increase was observed with serotonin antagonists and reuptake inhibitors (IRR:2.18, 95%CI 2.11–2.25). Subgroup analyses revealed higher prescription rates among older adults (65 + years; IRR:2.02, 95%CI 1.98–2.07) and those with higher CCI scores (4+; IRR:1.82, 95%CI 1.77–1.88). Sensitivity analysis confirmed a persistent increase in risk 1-year post-exposure, with IRR of 1.65 (95%CI 1.63–1.68) and 1.23 (95%CI 1.19–1.27) before and after 1 year, respectively.

**Conclusion:**

COVID-19 is significantly associated with an increased risk of antidepressant prescriptions, underscoring the need for enhanced mental health support and resources. Addressing stigma and ensuring timely interventions are essential for managing mental health in this context.

**Supplementary Information:**

The online version contains supplementary material available at 10.1186/s12888-025-07172-w.

## Highlights

This study found a significant association between SARS-CoV-2 infection and an increased risk of antidepressant prescription in Japan. - A higher risk of prescription was observed in older adults and in those with higher Charlson comorbidity index scores. Serotonin antagonists and reuptake inhibitors (SARIs) showed the largest increase in prescriptions.

**Supplementary Information:**

The online version contains supplementary material available at 10.1186/s12888-025-07172-w.

## Introduction

Depression has been recognized as a major mental health disorder reported during the COVID-19 pandemic [1–3], and multiple studies have reported a global increase in antidepressant prescriptions ranging from 11.6 to 18.5% compared to the pre-pandemic rate [4]. However, these increases may arise from diverse underlying factors, including increased clinical vigilance, potential overprescription, and increased burden of adverse effects among newly medicated patients. Therefore, there is a critical need to clarify how and why antidepressant prescriptions have changed in direct association with SARS-CoV-2 infection rather than conflating these trends with broader pandemic stressors.

Post-acute sequelae of COVID-19 (PASC) include fatigue, cognitive dysfunction (brain fog), and respiratory difficulties that can persist for several months [5, 6], and significantly impacts quality of life and overall health. The depressive status of PASC can reportedly persist for over a year, influenced by factors such as advanced age, male sex, high income, pre-existing health conditions, and psychosocial factors [7–9]. Long-term neurological or immunological alterations induced by the virus may continue to affect mental health, while persistent socioeconomic challenges or lifestyle changes resulting from the pandemic may contribute to ongoing mental health issues [10]. Consequently, some individuals may experience delayed manifestation of depressive symptoms. Persistent symptoms due to PASC impact the quality of life and daily functioning [11, 12]. Despite mounting evidence of increased antidepressant use during the pandemic, little is known about whether this heightened prescription rate is driven specifically by confirmed COVID-19 infection or by a general elevation in mental health needs across the population. Identifying the extent to which new or sustained antidepressant use is attributable to COVID-19 infection is essential for evaluating potential over-prescription, assessing long-term medication appropriateness, and understanding the risk–benefit profile of pharmacological interventions for individuals with persistent symptoms. This differentiation is crucial for evaluating potential overprescription, tailoring mental health interventions, and allocating resources efficiently in post-COVID healthcare systems.

While previous studies have reported an increase in depressive symptoms and antidepressant prescriptions following the COVID-19 pandemic, a significant knowledge gap remains regarding the long-term impact of SARS-CoV-2 infection on antidepressant use, particularly in Japan. In Japan, cultural and systemic factors further complicate pandemic-related mental health issues. Japan is characterized by high suicide rates and low utilization of psychiatric health services owing to societal stigma. Although benzodiazepine usage is high [13], strong stigma persists around antidepressants, and mental health services remain underutilized [14]. 

The implementation of social distancing measures, economic uncertainties, and disruptions to daily routines have contributed to an increase in depressive symptoms in the Japanese population. However, few studies have distinguished how SARS-CoV-2 infection specifically affects long-term antidepressant use. Previous research has also yielded inconsistent findings on the duration of post-pandemic prescription increases, some studies suggest a reversion to pre-pandemic levels within a few months, whereas others report sustained or even escalating utilization over a year or more [7, 15, 16] These discrepancies highlight gaps in understanding the clinical appropriateness of prescriptions, adverse event profiles, and the sociocultural barriers that can influence help-seeking behavior and medication persistence. Although the peak of the pandemic has passed, sporadic clusters of SARS-CoV-2 infection continue to be reported worldwide, including in Japan, underscoring that the pandemic has transitioned into an endemic phase rather than concluded outright [17]. Evidence from recent years shows enduring mental health sequelae of COVID-19, which merit continued scientific scrutiny [18, 19]. 

Japan’s elevated suicide rates, low psychiatric service uptake, and cultural stigmatization of psychiatric disorders underscore the importance of clarifying whether infection itself precipitates marked alterations in prescribing patterns [20]. By isolating infection status from pandemic-wide effects, our study sought to elucidate the mechanisms and contextual factors shaping antidepressant use and to inform targeted strategies for optimizing mental health care in both the acute and long-term phases of COVID-19 recovery.

We conducted a nationwide matched-cohort study using the Claims Database. Our objective was to measure both short- and long-term variations in the initiation of antidepressants following COVID-19 infection and to identify demographic and clinical factors that may influence these effects.

## Methods

### Research design and source

This matched cohort study used claims data. In this study, the National Insurance Claims Database between January 2015 and December 2022 was used to assess the pre-existing health status, COVID-19 infectious status, and new prescription of antidepressant drugs. The data source included outpatient and inpatient information stored at the individual level, allowing us to track patient-based visits and treatment.

### Study participants

Participants were individuals who had used the National Insurance Claims Database as of January 2020 in the five prefectures of Hiroshima, Kyoto, Osaka, Okayama, and Hyogo in Japan. The area covered 20.5 million population and approximately 16% of the population of Japan.

### Outcome

For this study, all antidepressant medications classified under the Anatomical Therapeutic Chemical (ATC) code “N60A” and available in Japan were assessed (Supplementary Table 1).The composite endpoint was initiation of antidepressant medications. The secondary endpoints were class-specific initiation of noradrenergic and specific serotonergic antidepressants (NaSSAs), serotonin-norepinephrine reuptake inhibitors (SNRIs), selective serotonin reuptake inhibitors (SSRIs), serotonin receptor inhibitors and modulators (SRIMs), tricyclic antidepressants (TCAs), tetracyclic antidepressants (TeCAs), and serotonin antagonists and reuptake inhibitors (SARIs). Psychotherapy initiation was set as a surrogate endpoint.

### Exposure

Participants confirmed to have COVID-19 during the study period were categorized as having exposure. COVID-19 patients required mandatory reporting during the study period and were exempted from the government’s medical expenses [21]. The public expense number on payment was used to ascertain exposure status.

### Data collection

Data were collected from the National Insurance Claims Database, including information on participants’ age category, sex, registered ICD-10 code for diseases, and prescription history. CCI was calculated using ICD-10 codes from the disease codes registered between 2015 and the enrollment month [22, 23]. The status of welfare is considered to be present when the payment for a claim is waived due to welfare or single-parent households.

### Data matching

The data were organized according to age category, sex, CCI total score, and month of enrollment in the insurance claims database (Supplementary Fig. 1). The month in which the participants were infected with COVID-19 was designated the index month. In the matching algorithm, we performed 1:1 exact matching of four factors: age category, sex, total CCI score, and month of insurance enrollment. After forming these matched pairs, we calculated the standardized mean differences (SMD) for each CCI category to assess the balance between groups for comorbidities and welfare status, ensuring that all SMD values were below 0.1. We excluded individuals who did not have a 1-year look-back period before the index month and those who had received any antidepressant drugs preceding the index month.

### Follow-up of the cohorts

All individuals in the matched cohort were followed up from the index date until the new prescription of antidepressants or the end of follow-up, whichever occurred first.

### Statistical analyses

All statistical analyses were performed using Stata Version 18 MP (StataCorp, TX, USA). Statistical significance was set at a p-value < 0.05.

Descriptive statistics were used to summarize the participants’ characteristics with or without SARS-CoV-2 infection status. For the main analysis, we used the Kaplan–Meier estimator to determine the cumulative incidence of the composite and secondary endpoint after the index date to estimate and visualize the cumulative incidence of antidepressant prescriptions over time for both groups. We assessed the discrepancy (per 1,000,000 person-months) and ratio of the cumulative incidences between patients with and without COVID-19, along with 95% confidence intervals for comparison. For subgroup analysis, we calculated the incidence rate ratio (IRR) of composite outcomes between the exposure and non-exposure groups for a subgroup of age, sex, and CCI categories. A sensitivity analysis was conducted to assess the robustness of the main analysis and subgroup analysis by splitting the study period into two intervals: the first 12 months after post-COVID-19 diagnosis and the period from 12 months or later.

### Ethical consideration and data availability

The study protocol was reviewed and approved by the Epidemiological Research Committee of Hiroshima University (approval number E2022-2025) and was conducted in accordance with the Declaration of Helsinki. The data that support the findings of this study are available from the Ministry of Health, Labor, and Welfare and are subject to strict confidentiality and privacy regulations (https://www.mhlw.go.jp/content/12400000/001158704.pdf, in Japanese). The requirement for informed consent to participate was waived because the data were obtained from an insurance claims database in an anonymous state.

## Results

Supplementary Fig. 2 shows a flowchart of this study. The study included approximately 16 million participants, with 6.1 million participants infected by COVID-19. The number of infected people in the five prefectures under study by the end of 2022 is reported by the Ministry of Health, Labour and Welfare to be 6.23 million [24], After excluding participants without matched cases and controls, approximately 2.5 million pairs were included.

The baseline characteristics of the matched cohort studies are presented in Table 1. The participants were predominantly female (55.1%). The most common age category is 70s. The numbers of CCI categories were 1,459,516 for CCI 0, 1,687,826 for CCI 1, 1,127,694 for CCI 2–3, and 853,538 for CCI 4 or higher. The selected comorbidities included acute myocardial infarction (AMI), congestive heart failure (CHF), cerebrovascular disease (CEVD), diabetes, liver disease (LD), renal disease (RD), cancer, and AIDS/HIV. Approximately 4.8% of the participants received welfare support. For the comorbidities listed, SMD values ranged from − 0.05 to 0.02, all remaining below 0.1.

**Table 1:** Baseline characteristics of study participants

	Total	Control group	COVID-19 group	
	*N* = 5,128,574	*N* = 2,564,287	*N* = 2,564,287	SMD
Female (%)	2,826,142 (55.1%)	1,413,071 (55.1%)	1,413,071 (55.1%)	0
Age category (%)				0
0–19	1,053,092 (21%)	526,546 (21%)	526,546 (21%)	
20–64	2,210,736 (43%)	1,105,368 (43%)	1,105,368 (43%)	
65 or over	1,864,746 (36%)	932,373 (36%)	932,373 (36%)	
CCI (%)				0
0	1,459,516 (28%)	729,758 (28%)	729,758 (28%)	
1	1,687,826 (33%)	843,913 (33%)	843,913 (33%)	
2–3	1,127,694 (22%)	563,847 (22%)	563,847 (22%)	
4 or over	853,538 (17%)	426,769 (17%)	426,769 (17%)	
Comorbidities (%)				
AMI	89,158 (1.7%)	44,335 (1.7%)	44,823 (1.7%)	−0.001
CHF	620,085 (12.1%)	299,637 (11.7%)	320,448 (12.5%)	−0.02
CEVD	653,688 (12.7%)	321,454 (12.5%)	332,234 (13.0%)	−0.01
Diabetes	345,313 (6.7%)	176,459 (6.9%)	168,854 (6.6%)	0.01
LD	1,066,858 (20.8%)	538,491 (21.0%)	528,367 (20.6%)	0.01
RD	184,915 (3.6%)	87,099 (3.4%)	97,816 (3.8%)	−0.02
Cancer	473,732 (9.2%)	243,516 (9.5%)	230,216 (9.0%)	0.02
AIDS	2,710 (0.1%)	1,567 (0.1%)	1,143 (0.0%)	0.007
Welfare usage (%)	246,245 (4.8%)	109,184 (4.3%)	137,061 (5.3%)	−0.05
Variants (time period)				0
Alpha	1,311,894 (25.6%)	655,947 (25.6%)	655,947 (25.6%)	
Delta	921,414 (18.0%)	460,707 (18.0%)	460,707 (18.0%)	
Omicron BA1/2	1,763,856 (34.4%)	881,928 (34.4%)	881,928 (34.4%)	
Omicron BA5	1,131,410 (22.1%)	565,705 (22.1%)	565,705 (22.1%)	

Data are presented as n (%) unless otherwise specified. SMD values < 0.1 indicate good balance between groups. The time period of each variant was set as alpha, from the start of o June 2021, delta, July to December 2021, Omicron BA1/2, January to June 2022, and Omicron BA5, July to December 2022

*CCI* Charlson comorbidity index, *AMI* Acute myocardial infarction, *CHF* Congestive heart failure, *CEVD* Cerebrovascular disease, *LD* Liver disease, *RD* Renal disease, *SMD* Standardized mean difference

**Fig. 1:**
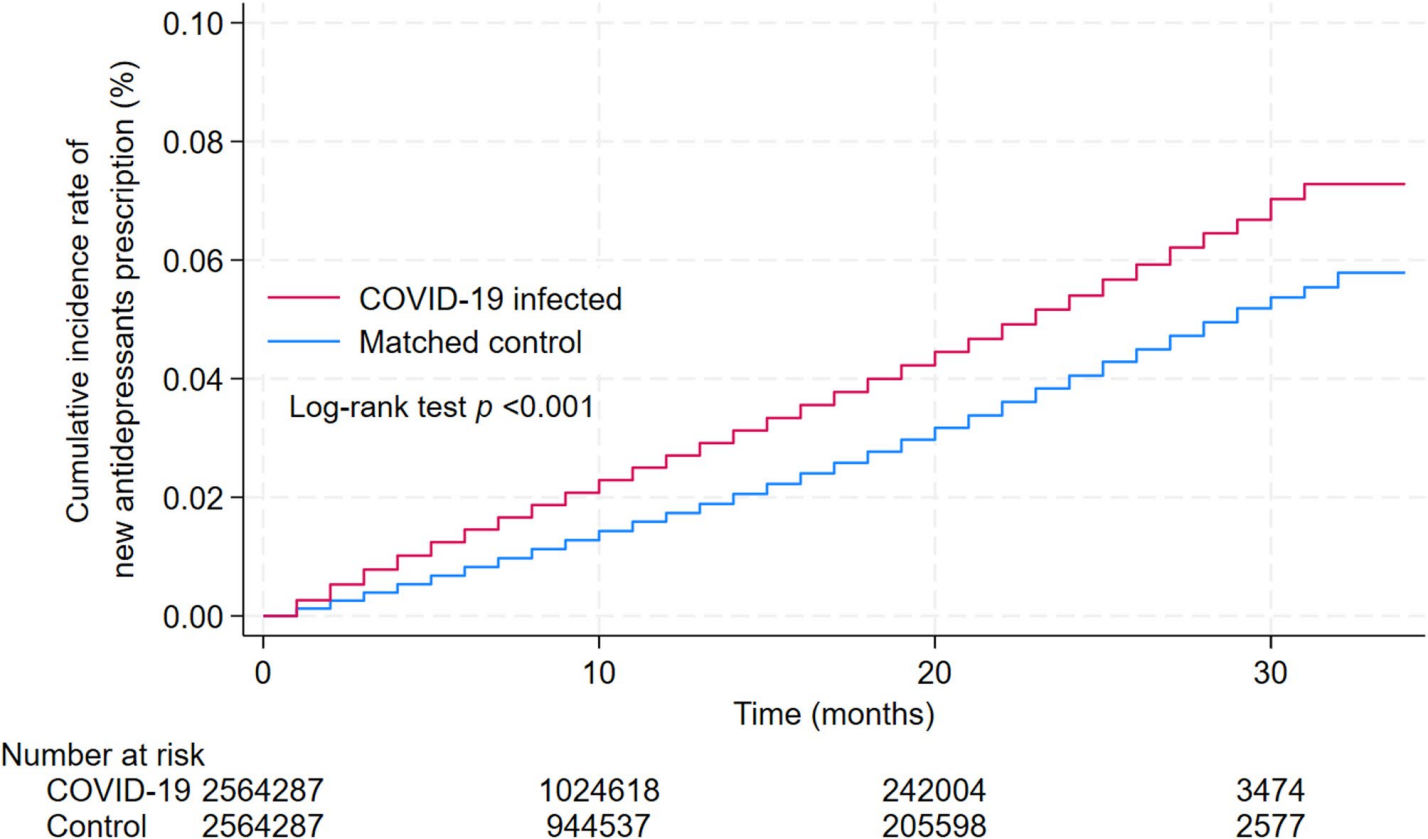
Kaplan–Meier analysis of new antidepressant prescription rates Cumulative incidence of new antidepressant prescriptions among individuals with COVID-19 (red line) and a matched non-infected control group (blue line). The p-value for the log-rank test was < 0.001, indicating a statistically significant difference between the two groups

Figure 1 shows the Kaplan–Meier curves for COVID-19 infected and matched controls for all participants. Table 2 shows the IRR and incidence rate difference (IRD) for the composite and secondary endpoints. The risk of prescribing antidepressants continuously increased during the study period. The study included 5,128,564 participants who were followed for a median duration of 7 months (interquartile range, 4–13 months). Of the 2,564,287 individuals in each group, 54,352 and 33,101 cases of the composite endpoint occurred in the COVID-19 infected and control groups, respectively, over 34 months after SARS-CoV-2 infection. The difference in cumulative incidence was 841 events/1,000,000 person-months (95% confidence interval [CI] 815–866), and the ratio of cumulative incidence was 1.56 (95% CI 1.54–1.58). The analyses of secondary endpoints showed an increased risk of prescription for any of the antidepressant categories in the COVID-19-infected group compared to the control group. During the study period, SSRIs and SNRIs were the most frequently prescribed; the incidence difference was 204 (95% CI 189–219) and 224 (95% CI 210–238) events per 1,000,000 person-months, and the incidence rate ratio was 1.38 (95% CI 1.34–1.41) and 1.47 (95% CI 1.44–1.51), respectively. SARIs were the 3rd most frequent prescriptions, with the highest incidence difference of 299 (95% CI 287–311) events per 1,000,000 person-months and an incidence ratio of 2.18 (95% CI 2.11–2.25) among secondary endpoints. The risk of new prescriptions increased significantly across other antidepressant categories. In surrogate endpoints, incidence difference of 2094 (95% CI 2044 to 2145) events per 1,000,000 person-months and an incidence ratio of 1.33 (95% CI 1.32–1.34).

**Table 2:** Number of events, incidence rate ratio, and incidence rate difference for composite and secondary endpoint

Event	Number of pairs	Events in COVID-19 Group	Events in Control Group	Cumulative Incidence (Events per 1 000 000 Person months)	
				*difference (95% CI)*	*ratio (95% CI)*
Composite endpoint	2564287	54352	33101	841 (815 to 866)	1.56 (1.54–1.58)
SSRIs	2564287	17387	11922	204 (189 to 219)	1.38 (1.34–1.41)
SNRIs	2564287	16412	10554	224 (210 to 238)	1.47 (1.44–1.51)
NaSSAs	2564287	6631	3363	131 (122 to 139)	1.86 (1.79–1.94)
TCAs	2564287	4865	3162	64.5 (56.8 to 72.1)	1.45 (1.39–1.52)
TeCAs	2564287	981	528	17.9 (14.6 to 21.2)	1.75 (1.58–1.95)
SRIMs	2564287	4315	3089	44.4 (37.0 to 51.7)	1.32 (1.26–1.38)
SARIs	2564287	12993	5646	299 (287 to 311)	2.18 (2.11–2.25)
Surrogate endpoint					
Psychotherapy	2564287	1914115	139177	2094 (2044 to 2145)	1.33 (1.32–1.34)

*SSRIs* Selective serotonin reuptake inhibitors, *SNRIs* Serotonin-norepinephrine reuptake inhibitors, *NaSSAs* Noradrenergic and specific serotonergic antidepressants, *TCAs* Tricyclic antidepressants, *TeCAs* Tetracyclic antidepressants, *SRIMs* Serotonin receptor inverse agonists, *SARIs* Serotonin antagonist reuptake inhibitors

**Fig. 2:**
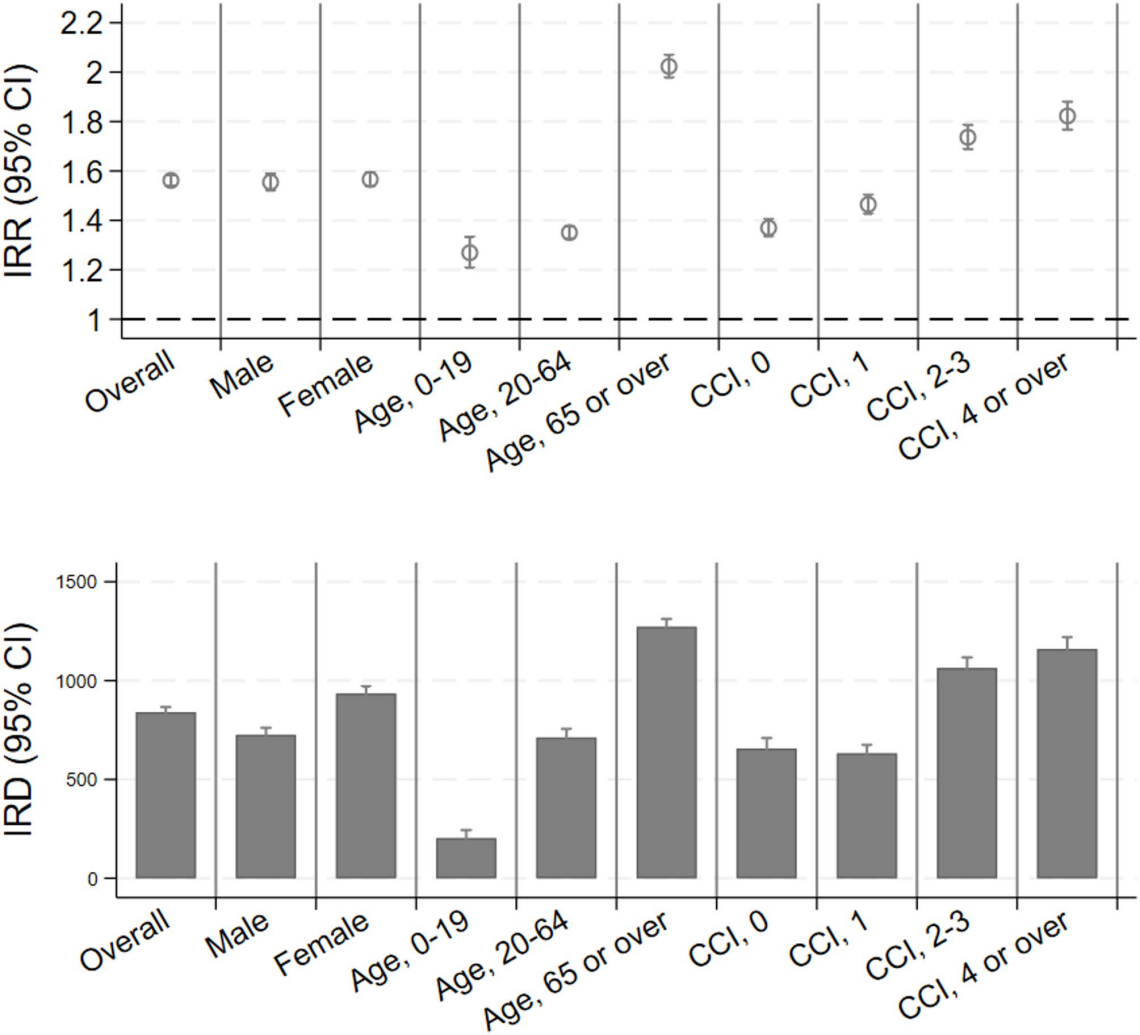
IRR and IRD for each category of subgroup. Incidence rate ratios of categories in terms of age, sex, and Charlson comorbidity index were plotted for all periods. The number of participants and new prescriptions were reported. The actual numbers are listed in Supplementary Table 2. IRR, incidence rate ratio; IRD, incidence rate difference; CCI, Charlson comorbidity index; CI, confidence interval

Figure 2 and Supplementary Table 2 show the IRRs and IRDs of antidepressant prescriptions in the COVID-19-infected group compared to the control group in all subgroups. According to the data, individuals aged 65 years and above (2.02, 95% CI 1.98–2.07) and those with higher CCI scores (1.82, 95% CI 1.77–1.88) had a higher incidence rate of antidepressant prescriptions than those aged < 20 years (1.27, 95% CI 1.21–1.33) and those with lower CCI scores (1.37, 95% CI 1.34–1.41). In terms of sex, the ratio was comparable between males (1.56, 95% CI 1.52–1.59) and females (1.57, 95% CI 1.54–1.59).

**Fig. 3:**
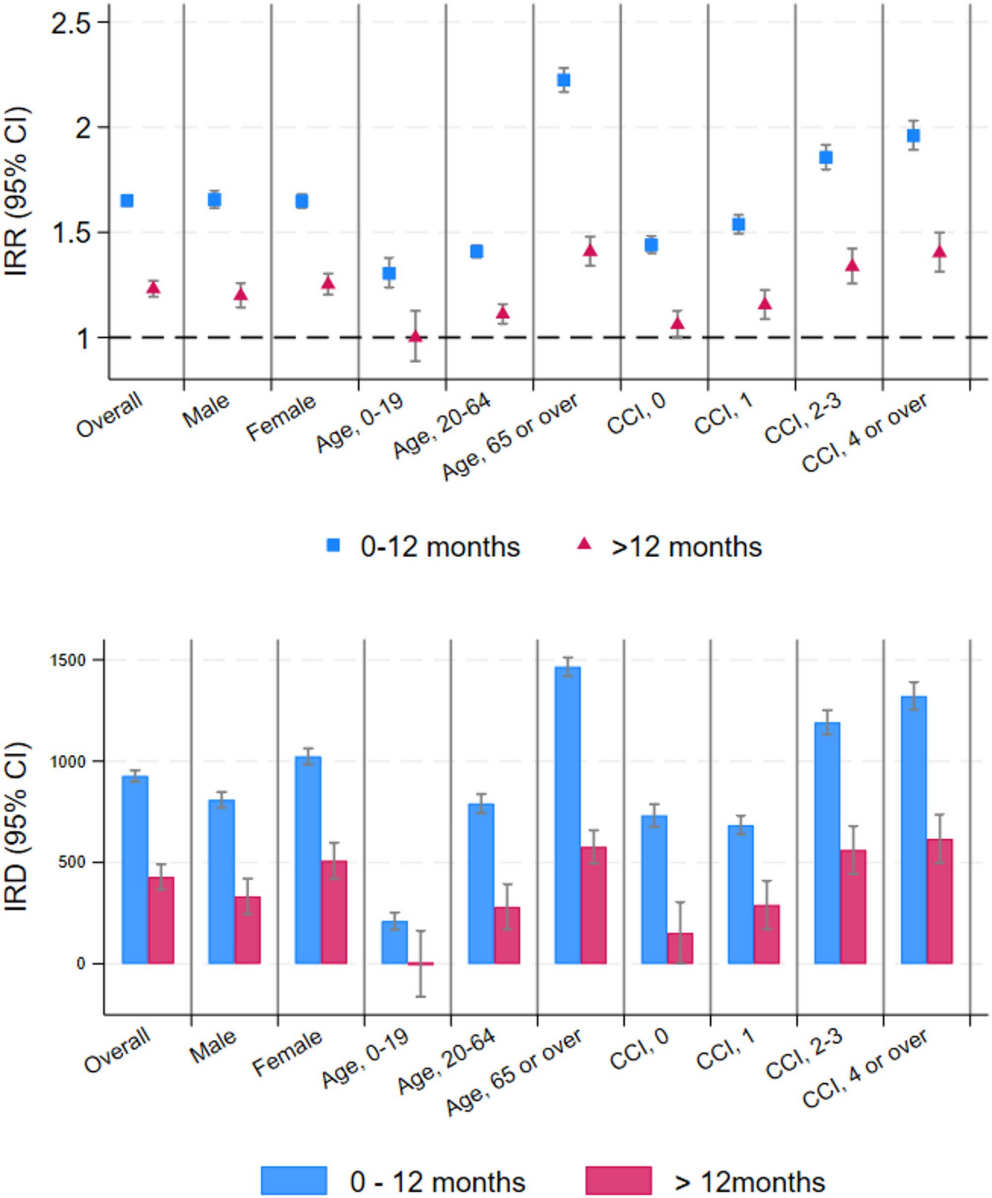
IRR and IRD of sensitivity analysis. Subgroup analyses for a separated follow-up period of ≤ 1 year (blue) and > 1 year (red). IRR and IRD are shown with their 95% CIs. Detailed numbers are presented in Supplementary Table 3. IRR, incidence rate ratio; IRD, incidence rate difference; CCI, Charlson comorbidity index; CI, confidence interval

The sensitivity analysis in Fig. 3 and Supplementary Table 3 demonstrates the IRRs and IRDs for the composite endpoint and subgroup categories, both within and after 1 year. The incidence risk ratio within the 1 year was 1.65 (95% CI 1.63–1.68), whereas the IRR after 1 year was 1.23 (95% CI 1.19–1.27). In all subgroups, the risk within 1 year was higher than that after 1 year. Additionally, the risk ratio was greater than 1 in all subgroups for analyses within 1 year. In the analyses of participants after a 1-year follow-up period, the risk ratio was greater than 1 in all subgroups, except the 0–19 years subgroup (IRR 1.00, 95% CI 0.88 − 1.12).

## Discussion

Our findings indicate that the risk of prescribed antidepressants increased approximately 1.6 times in patients with COVID-19 compared with those without COVID-19. Subgroup analyses revealed that older age and higher CCI scores were associated with a higher risk of antidepressant prescription. The increased risk of antidepressant prescription continued throughout the study period.

In contextualizing our findings, it is important to note the conclusions from recent landmark studies that examined the psychiatric consequences of COVID-19. A study that followed US veterans for three years found an increased risk of developing numerous diseases during the pandemic, suggesting broader pandemic-related stressors [19]. Studies using Japanese and South Korean data also noted an increased risk of psychiatric disorders beyond one year of frequency [25]. These studies relied on diagnostic codes to determine the outcomes and the validity of these codes might be uncertain in mental disorders. By measuring actual dispensing, our study demonstrates that the clinical response as a new antidepressant treatment has also remained elevated, thereby providing pharmaco-epidemiological confirmation of earlier diagnostic findings. A nested case-control study in Italy suggested a 1.5-times higher likelihood of antidepressant prescriptions following a COVID- 19 infection [26], however, the study did not quantify the absolute excess risk. Our risk difference of approximately 900 prescriptions per million person-months translates these relative effects into concrete implications for resources. Finally, a recent U.S. primary-care cohort found no correlation between COVID-19 and antidepressant prescriptions [27]. This variation might indicate differing perspectives on health-seeking behaviors among individuals in primary care registries. In Japan, there is a tendency for a higher threshold for psychiatric referrals, which makes dispensing data a more sensitive measure of unmet needs. Taken together, these comparisons underline the persistent, infection-specific pharmacologic burden revealed in our nationwide cohort and underscore the necessity of post-COVID medication stewardship in high-suicide-rate settings such as Japan.

Among the secondary endpoints, the largest increase was observed in SARIs. When each prescription drug group was examined by age, the IRRs for SARIs and NASSAs after COVID-19 were higher in the older individuals than in the control group (2.74 [95% CI 2.63–2.86] and 2.51 [95% CI 2.34–2.70], respectively; Supplementary Table 4). Depression likely involves a combination of cognitive impairment, sleep disturbance, and immune dysregulation [22, 28, 29]. In this study, the number of prescriptions for SARI and NaSSA, which are also used for sleep disorders, increased, suggesting that the number of prescriptions for these drugs increased with concomitant sleep disorders [30, 31]. SARIs are often used to treat delirium, and older individuals can become delirious with SARS-CoV-2 infection, which may have led to an increase in new prescriptions [32–34]. We posit that these prescribing patterns align with Japanese clinical practice. Guidelines for the management of depression in older individuals in Japan do not clearly indicate first-line agents [35]; antidepressants other than TCAs are recommended from the viewpoint of side effect profiles. In this study, TCAs were the least frequently administered drugs, except for less frequently used TeCAs. TCA is administered more frequently to younger patients than other drugs, and although it is not covered by insurance, the possibility that it is administered for prophylactic purposes, such as migraine headaches, should be considered [36, 37].

The upward trajectory of antidepressant use that we observed in both SARS-CoV-2–positive participants and their matched controls is consistent with 2 year trend. International surveillance shows that antidepressant consumption rose by nearly 50% between 2011 and 2021 and by a further 10% between 2019 and 2021, even in the absence of infection-specific effects [38–40]. Several pandemic-wide forces likely accelerated this trajectory: (i) pervasive economic and social stress, (ii) curtailed access to psychotherapy prompting a pharmacological fallback, and (iii) Japan’s emergency relaxation of telemedicine rules, which from April 2020 permitted remote first visits and was formalized in January 2022 [41]. A recent nationwide utilization study further reported that, while the number of psychiatric visits dipped after the first state of emergency, the average length of each prescription increased, implying a shift toward longer-term drug management [18]. These secular influences explain the parallel rise across cohorts, whereas the excess risk quantified in our study isolates the portion specifically attributable to a confirmed infection.

Subgroup analyses aimed to determine the influence of demographic and health status variables on the risk of antidepressant prescriptions post-SARS-CoV-2 infection and revealed an increased risk among older individuals and higher CCI scores. Older individuals may be more susceptible to the psychological impacts of COVID-19 owing to isolation, higher morbidity, and mortality fears and are at an increased risk of developing depression [42]. These findings underscore the need for targeted mental health interventions, particularly those designed for older populations and for individuals with significant comorbidities. The frequency of psychotherapy administered in conjunction with medication has also increased after COVID-19, reflecting the increased demand for psychiatric care. Tailoring the support mechanisms for these vulnerable groups could mitigate the heightened risk of depression, necessitating antidepressant prescription.

Our sensitivity analysis, which assessed the risk variations within and beyond 1-year post-infection, further corroborates the robustness of our subgroup findings. However, in this study, while a trend toward improvement was observed, it also revealed a sustained elevated risk, emphasizing the long-term impact of COVID-19 on mental health across these specific subgroups. Besides, previous studies suggest that the impact of COVID-19 may differ depending on the circulating variant [43]. Accordingly, we performed an additional analysis for each variant (Supplementary Table 5). While the results showed a significantly elevated risk across all variants, direct comparisons are challenging because each variant emerged under different pandemic conditions and over different observation periods. Further studies are needed to determine the prognoses of the early- and late-onset groups and infected group for different variants.

Multiple biological processes have been implicated in depressive symptoms of PASC, including immune system dysregulation, cerebral microvasculature thrombosis, neuroanatomical changes, and drug adverse effects of treatment [9, 44, 45]. In Japan, these sequelae coincide with a documented rise in mental-health consultations and suicide rates during the pandemic [20, 46]. This situation is further exacerbated by the cultural stigma associated with seeking medical help for psychological symptoms, a factor that may deter many from accessing necessary care [47–49].

To mitigate this burden, we propose a three-pronged response. First, national campaigns to destigmatize mental health concerns should be launched to encourage individuals to seek help promptly. Such campaigns could play a crucial role in altering public perceptions, thereby reducing the stigma surrounding antidepressant prescriptions and facilitating early interventions. Second, integrating mental health screenings into the standard post-COVID-19 follow-up process could aid in the early detection of mental health issues and ensure timely referrals to psychiatric services. Finally, policy reforms focusing on the mental health aftermath of pandemics are essential. These reforms should include a substantial allocation of resources for mental health services and the incorporation of mental health support into emergency response plans, ultimately fostering a more resilient healthcare system capable of addressing the complexities of post-pandemic mental health care.

A strength of this study is that it used a National Insurance Claims Database, including 2.5 million exposure and non-exposed individuals. Second, the certainty of the confirmation of SARS-CoV-2 infection would be accurate. Additionally, the database is paid for by public funds; hence, a reimbursement code has been established for the treatment of patients diagnosed with COVID-19, and during the study period, all the SARS-CoV-2 infections were mandatorily reported in Japan. Third, this study used first-time antidepressant prescriptions as the study outcome, which may enhance methodological rigor. This approach minimizes biases associated with prevalent user effects, which can confound outcomes owing to differences in disease duration, severity, or previous treatment exposures. By focusing on new prescriptions, we ensured a more accurate assessment of the impact of COVID-19 on antidepressant use. Furthermore, the exclusion of prevalent users from the study cohort enhances the robustness of our findings, as it eliminates potential biases from individuals already on antidepressant therapy who may have different baseline mental health statuses compared to the general population. This methodological choice allows for a clearer understanding of the direct influence of COVID-19 on antidepressant prescription patterns, free from the confounding effects of ongoing treatments and pre-existing conditions.

This study has certain limitations. A limitation of this study is the lack of data on the severity of COVID-19, which may have influenced the risk of antidepressant prescription. Second, selection bias due to misclassification of SARS-CoV-2 infection status cannot be avoided. Some individuals who had SARS-CoV-2 infection with mild symptoms and no diagnostic test performed may have been classified as the non-exposure group, although this bias may have worked as a point estimate into null. We contend that the influence of this bias is minimal in Japan, where COVID-19 is classified as an all-count disease, and stringent measures were enforced until May 2023, when the classification under the Infectious Disease Control Law was revised. Notably, Japan was among the countries with the lowest infection rates during the initial stages of the pandemic. Third, we did not match other potential social factors, including vaccination status, economic status, and employment status. Although we included individuals from five prefectures, we could not match by region or income level as the dataset did not contain sufficient granularity on socioeconomic factors. We did not have vaccination data; therefore, we could not evaluate how vaccination status modifies the risk of new antidepressant prescriptions. In addition, this study could not ascertain the presence or absence of death as a competing risk, which may have resulted in underestimation of the original risk. However, the potential influence of this factor cannot be dismissed. Fourth, patients infected closer to December 2022 had inherently shorter follow-up periods, which may overestimate antidepressant risk by capturing events early but not allowing sufficient time for others to develop. Researchers in future studies should investigate the impact of COVID-19 on antidepressant prescriptions across diverse healthcare systems and cultural contexts, including healthcare accessibility, prescription practices, and societal attitudes towards mental health and pharmacological interventions. Such studies could provide more generalizable insights and elucidate specific regional or national challenges and opportunities in managing mental health during and after pandemics.

## Conclusions

This large-scale matched cohort study provides the evidence of a potential association between SARS-CoV-2 infection and increased antidepressant prescriptions in Japan, with the elevated risk persisting beyond 1 year post-infection, particularly among older adults and those with higher comorbidity burdens. These findings underscore the long-term impact of COVID-19 on mental health and highlight the need for enhanced support, especially for vulnerable populations. The observed increase in SARI and NaSSA prescriptions suggests potential sleep disturbances and delirium as contributing factors, particularly in older patients.

To address these challenges, a multifaceted approach is necessary. This approach must include destigmatization campaigns, integration of mental health screenings into post-COVID follow-up care, and policy reforms to allocate resources for mental health services. Healthcare providers should remain vigilant for signs of depression, even months after initial infection, and be prepared to offer appropriate interventions. By proactively addressing the mental health sequelae of COVID-19, we can mitigate its long-term impact and improve overall public health outcomes in the post-pandemic era.

## Supplementary Information

Supplementary Material 1.

## Data Availability

The data that support the findings of this study are available from the Ministry of Health, Labor, and Welfare and are subject to strict confidentiality and privacy regulations (https://www.mhlw.go.jp/content/12400000/001158704.pdf, in Japanese).

## Abbreviations

PASC: Post-Acute Sequelae of SARS-CoV-2 infection
CCI: Charlson Comorbidity Index
IRR: Incidence Rate Ratio
IRD: Incidence Rate Difference
ICD-10: International Statistical Classification of Diseases and Related Health Problems, 10th Revision
ATC: Anatomical Therapeutic Chemical
NaSSAs: Noradrenergic and Specific Serotonergic Antidepressants
SNRIs: Serotonin-Norepinephrine Reuptake Inhibitors
SSRIs: Selective Serotonin Reuptake Inhibitors
SRIMs: Serotonin Receptor Inhibitors and Modulators
TCAs: Tricyclic Antidepressants
TeCAs: Tetracyclic Antidepressants
SARIs: Serotonin Antagonists and Reuptake Inhibitors
CI: Confidence Interval
