# Supplementary material for: Impact of COVID-19 on antidepressant prescription: a matched cohort study using the National insurance claims database in Japan
Source: BMC Psychiatry. 2025 Jul 25;25:725. doi: 10.1186/s12888-025-07172-w (PMC12291366; doi:10.1186/s12888-025-07172-w)
Supplement: Supplementary file 1 — Supplementary Material 1. [file 12888_2025_7172_MOESM1_ESM.docx]

**Supplementary Figure 1.** Illustration of study design

In the matched cohort design, each COVID-19-infected individual was matched for sex, age, CCI total score, and enrollment month of health insurance. Follow-up began at the index month. Individuals were followed until the event of interest or the end of the observational period.

CCI, Charlson comorbidity index


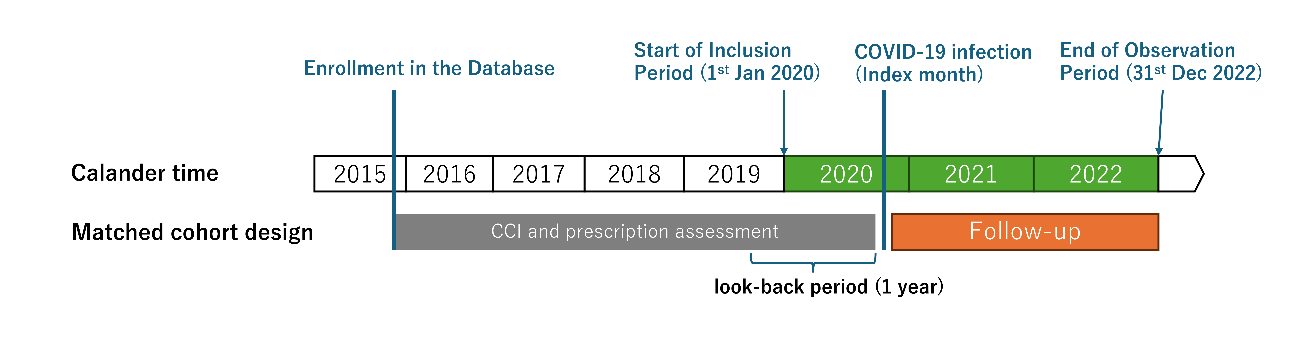


**Supplementary Figure 2.** Flow chart of study participants

Approximately 6 million individuals out of a total of approximately 16 million participants were found to be infected. Following the exclusion of 3,122,771 participants without any follow-up period, 104,095 participants without a 1-year look-back period and 9,800 participants who had received antidepressant drugs previously, a total of 2,869,680 participants were deemed eligible for the exposure group and matched cohort study. Finally, 2,564,287 pairs were matched and analyzed. In the matching process, age categories were divided as every 1 year for participants under 10 years old and in increments of 5 years for participants aged 10 years and older. The Charlson comorbidity index was matched using total scores.


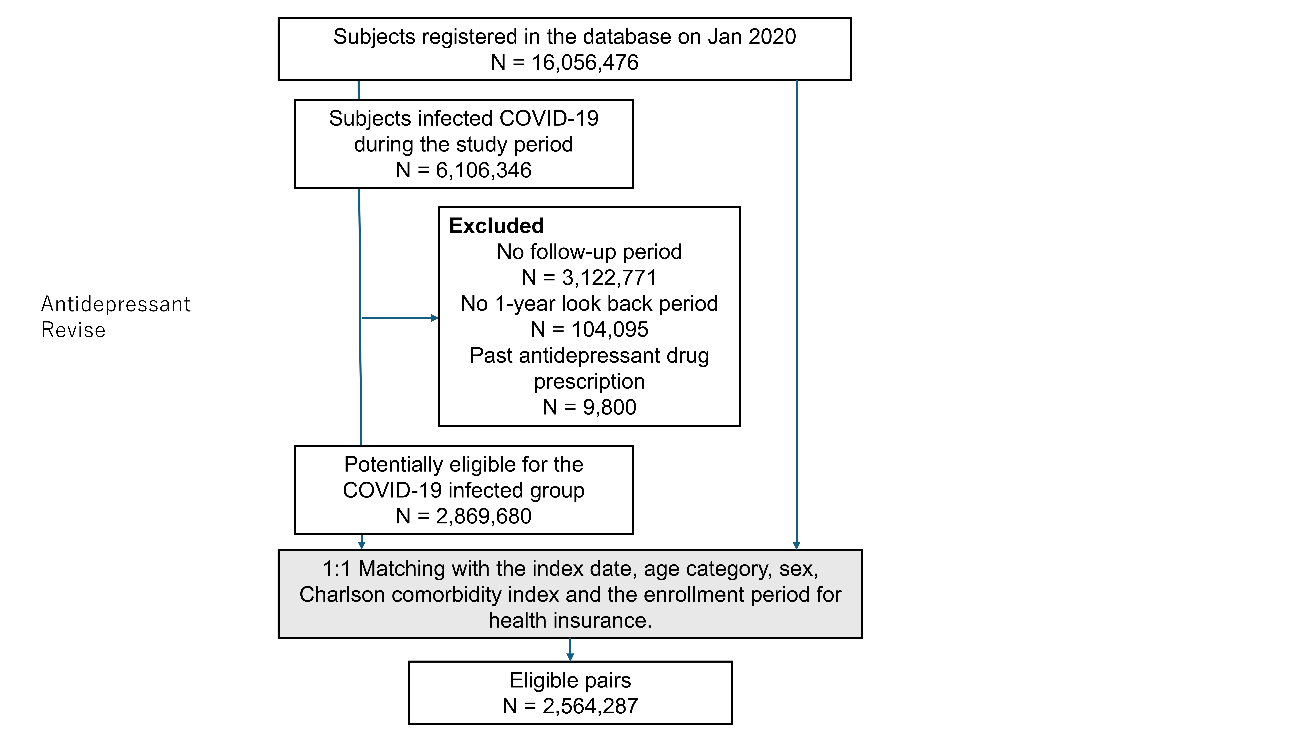


**Supplementary Table 1**

| ATC code | Antidepressants Category | Drugs |
| --- | --- | --- |
| N06AA | Tricyclic Antidepressants (TCAs) | Imipramine, Clomipramine, Amitriptyline, Nortriptyline, Doxepin, Protriptyline, Amoxapine |
|  | Tetracyclic Antidepressants (TeCAs) | Maprotiline, Mianserin, Setiptiline |
| N06AB | Selective Serotonin Reuptake Inhibitors (SSRIs) | Fluvoxamine, Sertraline, Escitalopram, Paroxetine |
| N06AX | Serotonin-Norepinephrine Reuptake Inhibitors (SNRIs) | Duloxetine, Venlafaxine, Milnacipran |
|  | Noradrenergic and Specific Serotonergic Antidepressants (NaSSAs) | Mirtazapine |
|  | Serotonin Receptor Modulators and Inhibitors (SRMs) | Vortioxetine |
|  | Serotonin Antagonists and Reuptake Inhibitors (SARIs) | Trazodone |

ATC, Anatomical Therapeutic Chemical

| **Supplementary Table 2.** Number of events, incidence rate ratio, and incidence rate difference for subgroup categories | | | | | |  |  |
| --- | --- | --- | --- | --- | --- | --- | --- |
| **Subgroup** | **No. of Subjects** | **No. of Events in the COVID-19 infected Group** | **No. of Events in the Control Group** | **Cumulative Incidence (No. of Events per 1 000 000 Person months)** | | |  |
|  |  |  |  |  |  |  |  |
|  |  |  |  |  |  |  |  |
|  |  |  |  | ***Difference (95% CI)*** | ***Ratio (95% CI)*** | |  |
| Male | 2302432 | 21394 | 13105 | 726 (691 to 761) | 1.55 (1.52–1.59) | |  |
| Female | 2826142 | 32958 | 19996 | 935 (899 to 971) | 1.57 (1.54–1.59) | |  |
| Age, 0–19 | 1053092 | 3967 | 2742 | 203 (162 to 244) | 1.27 (1.21–1.33) | |  |
| Age, 20–64 | 2210736 | 27831 | 19029 | 712 (669 to 756) | 1.35 (1.33–1.38) | |  |
| Age, 65 or over | 1864746 | 22554 | 11330 | 1272 (1232 to 1312) | 2.02 (1.98–2.07) | |  |
| CCI, 0 | 1459516 | 14748 | 10027 | 657 (604 to 709) | 1.37 (1.34–1.41) | |  |
| CCI, 1 | 1687826 | 14667 | 9216 | 632 (589 to 674) | 1.46 (1.43–1.5) | |  |
| CCI, 2–3 | 1127694 | 13655 | 7605 | 1064 (1011 to 1117) | 1.74 (1.69–1.79) | |  |
| CCI, 4 or over | 853538 | 11282 | 6253 | 1160 (1101 to 1219) | 1.82 (1.77–1.88) | |  |

CCI, Charlson comorbidity index; CI, confidence interval

**Supplementary Table 3.** Number of events, incidence rate ratio, and incidence rate difference for sensitivity analysis

| Subgroup | Analysis Period | No. of Subjects | No. of Events in COVID-19 infected Group | No. of Events in Control Group | Cumulative Incidence (No. of Events per 1 000 000 Person months) | |  |
| --- | --- | --- | --- | --- | --- | --- | --- |
|  |  |  |  |  | Difference (95% CI) | Ratio (95% CI) |  |
| Overall | <=12m | 5128574 | 44426 | 26093 | 927 (899 to 954) | 1.65 (1.63–1.68) |  |
| Overall | >12m | 1313775 | 9926 | 7008 | 429 (366 to 491) | 1.23 (1.19–1.27) |  |
| Male | <=12m | 2302432 | 17419 | 10216 | 809 (770 to 847) | 1.66 (1.62–1.7) |  |
| Male | >12m | 597847 | 3975 | 2889 | 333 (245 to 420) | 1.20 (1.14–1.26) |  |
| Female | <=12m | 2826142 | 27007 | 15877 | 1023 (983 to 1063) | 1.65 (1.62–1.68) |  |
| Female | >12m | 715928 | 5951 | 4119 | 508 (419 to 597) | 1.25 (1.20–1.3) |  |
| Age, 0–19 | <=12m | 1053092 | 3289 | 2274 | 211 (169 to 252) | 1.31 (1.24–1.38) |  |
| Age, 0–19 | >12m | 162246 | 678 | 468 | -1 (-163 to 162) | 1.00 (0.89–1.13) |  |
| Age, 20–64 | <=12m | 2210736 | 22480 | 15238 | 790 (743 to 837) | 1.41 (1.38–1.44) |  |
| Age, 20–64 | >12m | 556799 | 5351 | 3791 | 281 (170 to 392) | 1.11 (1.06–1.16) |  |
| Age, 65 or over | <=12m | 1864746 | 18657 | 8581 | 1465 (1419 to 1511) | 2.22 (2.17–2.28) |  |
| Age, 65 or over | >12m | 594730 | 3897 | 2749 | 577 (495 to 658) | 1.41 (1.34–1.48) |  |
| CCI, 0 | <=12m | 1459516 | 12178 | 8079 | 731 (676 to 787) | 1.44 (1.40–1.48) |  |
| CCI, 0 | >12m | 303669 | 2570 | 1948 | 152 (2 to 303) | 1.06 (1.00–1.13) |  |
| CCI, 1 | <=12m | 1687826 | 12002 | 7373 | 684 (639 to 730) | 1.54 (1.49–1.58) |  |
| CCI, 1 | >12m | 376615 | 2665 | 1843 | 290 (171 to 408) | 1.15 (1.09–1.23) |  |
| CCI, 2-3 | <=12m | 1127694 | 11129 | 5910 | 1191 (1131 to 1251) | 1.86 (1.80–1.92) |  |
| CCI, 2–3 | >12m | 335595 | 2526 | 1695 | 561 (444 to 679) | 1.34 (1.26–1.42) |  |
| CCI, 4 or over | <=12m | 853538 | 9117 | 4731 | 1321 (1253 to 1389) | 1.96 (1.89–2.03) |  |
| CCI, 4 or over | >12m | 297896 | 2165 | 1522 | 617 (498 to 736) | 1.40 (1.31–1.50) |  |

>12m, more than 12 months; <=12m, 12 month or less; CCI, Charlson comorbidity index; CI, confidence interval

| Supplemental Table 4. Number of events, incidence rate ratio, and incidence rate difference for composite and secondary endpoint among age categories. | | | | | | |  |  |
| --- | --- | --- | --- | --- | --- | --- | --- | --- |
|  | Age Category (years) | Number of Subjects | Events in the COVID-19 Group | Events in the Control Group | Cumulative Incidence (Events per 1 000 000 Person months) | | |  |
|  |  |  |  |  | difference (95% CI) | ratio (95% CI) | |  |
| SSRIs | 0–19 | 1053092 | 2287 | 1723 | 78 (46 to 110) | 1.16 (1.09–1.24) | |  |
|  | 20–64 | 1864746 | 11967 | 8617 | 256 (228 to 285) | 1.28 (1.24–1.31) | |  |
|  | 65 or over | 5128574 | 3004 | 1497 | 169 (154 to 183) | 2.03 (1.9–2.16) | |  |
| SNRIs | 0–19 | 1053092 | 519 | 340 | 32 (17 to 46) | 1.34 (1.16–1.54) | |  |
|  | 20–64 | 2210736 | 7737 | 5144 | 210 (188 to 233) | 1.38 (1.34–1.43) | |  |
|  | 65 or over | 1864746 | 8006 | 4966 | 345 (320 to 370) | 1.63 (1.57–1.69) | |  |
| NaSSAs | 0–19 | 1053092 | 319 | 194 | 23 (12 to 35) | 1.44 (1.2–1.73) | |  |
|  | 20–64 | 2210736 | 3453 | 2011 | 124 (109 to 138) | 1.58 (1.49–1.67) | |  |
|  | 65 or over | 1864746 | 2789 | 1120 | 186 (173 to 200) | 2.51 (2.34–2.7) | |  |
| TCAs | 0–19 | 1053092 | 723 | 413 | 61 (44 to 77) | 1.53 (1.36–1.74) | |  |
|  | 20–64 | 2210736 | 2663 | 1772 | 72 (58 to 85) | 1.38 (1.3–1.47) | |  |
|  | 65 or over | 1864746 | 1439 | 948 | 55 (45 to 66) | 1.53 (1.41–1.66) | |  |
| TeCAs | 0–19 | 1053092 | 55 | 30 | 5 (0 to 10) | 1.61 (1.01–2.6) | |  |
|  | 20–64 | 2210736 | 483 | 281 | 17 (12 to 23) | 1.58 (1.36–1.83) | |  |
|  | 65 or over | 1864746 | 436 | 212 | 25 (20 to 31) | 2.07 (1.76–2.46) | |  |
| SRIMs | 0–19 | 1053092 | 412 | 293 | 19 (5 to 32) | 1.23 (1.06–1.44) | |  |
|  | 20-64 | 2210736 | 3357 | 2496 | 62 (47 to 78) | 1.24 (1.17–1.3) | |  |
|  | 65 or over | 1864746 | 515 | 272 | 27 (21 to 33) | 1.91 (1.65–2.22) | |  |
| SARIs | 0–19 | 1053092 | 519 | 340 | 44 (31 to 56) | 1.71 (1.45–2.02) | |  |
|  | 20–64 | 2210736 | 7737 | 5144 | 166 (150 to 182) | 1.67 (1.59–1.76) | |  |
|  | 65 or over | 1864746 | 8006 | 4966 | 584 (561 to 607) | 2.74 (2.63–2.86) | |  |

SSRIs, selective serotonin reuptake inhibitors; SNRIs: serotonin-norepinephrine reuptake inhibitors; NaSSAs: noradrenergic and specific serotonergic antidepressants; TCAs: tricyclic antidepressants; TeCAs: tetracyclic antidepressants; SRIMs: serotonin receptor inverse agonists; SARIs: serotonin antagonist reuptake inhibitor; CI: confidence interval

| **Supplementary Table 5.** Number of events, incidence rate ratio, and incidence rate difference for composite and secondary endpoint | | | | | |  |  |
| --- | --- | --- | --- | --- | --- | --- | --- |
| **Variant** | **Number of pairs** | **Events in COVID-19 Group** | **Events in Control Group** | **Cumulative Incidence (Events per 1 000 000 Person months)** | | |  |
|  |  |  |  |  |  |  |  |
|  |  |  |  |  |  |  |  |
|  |  |  |  | ***difference (95% CI)*** | ***ratio (95% CI)*** | |  |
| Alpha | 1,311,894 | 23935 | 13866 | 864 (828 to 900) | 1.64 (1.60–1.67) | |  |
| Delta | 921,414 | 12169 | 6882 | 970 (914 to 1026) | 1.65 (1.61–1.70) | |  |
| Omicron BA1/2 | 1,763,856 | 13601 | 9212 | 678 (627 to 729) | 1.42 (1.38–1.46) | |  |
| Omicron BA5 | 1,131,410 | 4647 | 3141 | 912 (804 to 1021) | 1.46 (1.39–1.53) | |  |

The time period of each variant was set as Alpha, from the start of o June 2021, Delta, July to December 2021, Omicron BA1/2, January to June 2022, and Omicron BA5, July to December 2022.
